# Supplementary material for: Identification of the Prognostic Biomarkers CBX6 and CBX7 in Bladder Cancer
Source: Diagnostics (Basel). 2023 Apr 11;13(8):1393. doi: 10.3390/diagnostics13081393 (PMC10137658; doi:10.3390/diagnostics13081393)
Supplement: Supplementary file 1 [file diagnostics-13-01393-s001.zip › diagnostics-2296881-supplementary.pdf]

Identification of the prognostic biomarkers CBX6 and CBX7 in bladder cancer

Supplementary Figures

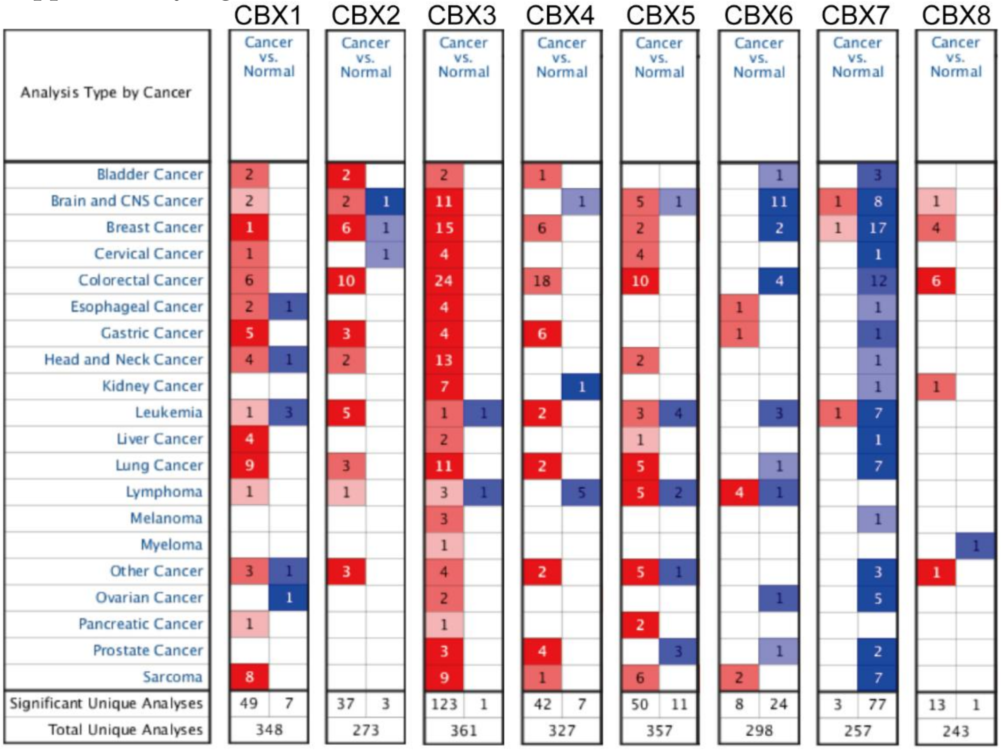

Analysis Type by Cancer	CBX1 Cancer vs. Normal		CBX2 Cancer vs. Normal		CBX3 Cancer vs. Normal		CBX4 Cancer vs. Normal		CBX5 Cancer vs. Normal		CBX6 Cancer vs. Normal		CBX7 Cancer vs. Normal		CBX8 Cancer vs. Normal	
Bladder Cancer	2		2		2		1					1		3		
Brain and CNS Cancer	2		2	1	11			1	5	1		11	1	8	1	
Breast Cancer	1		6	1	15		6		2			2	1	17	4	
Cervical Cancer	1			1	4				4					1		
Colorectal Cancer	6		10		24		18		10			4		12	6	
Esophageal Cancer	2	1			4						1			1		
Gastric Cancer	5		3		4		6				1			1		
Head and Neck Cancer	4	1	2		13				2					1		
Kidney Cancer					7			1						1	1	
Leukemia	1	3	5		1	1	2		3	4		3	1	7		
Liver Cancer	4				2				1					1		
Lung Cancer	9		3		11		2		5			1		7		
Lymphoma	1		1		3	1		5	5	2	4	1				
Melanoma					3									1		
Myeloma					1											1
Other Cancer	3	1	3		4		2		5	1				3	1	
Ovarian Cancer		1			2							1		5		
Pancreatic Cancer	1				1				2							
Prostate Cancer					3		4			3		1		2		
Sarcoma	8				9		1		6		2			7		
Significant Unique Analyses	49	7	37	3	123	1	42	7	50	11	8	24	3	77	13	1
Total Unique Analyses	348		273		361		327		357		298		257		243	

Figure S1. Transcriptional expression comparison of CBXs in different types of cancers analyzed by ONCOMINE database. mRNA level was compared by student's *t*-test. Threshold setting: P value < 0.01; fold change: 1.5; gene rank: 10%.

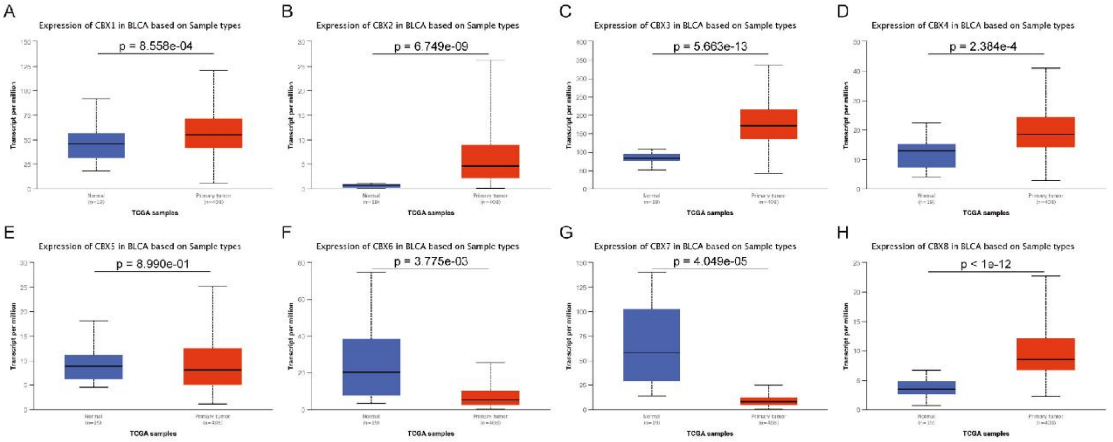

Figure S2. Expression levels of CBXs mRNA in BLCA and normal tissues analyzed by UALCAN database. (A) CBX1. (B) CBX2. (C) CBX3. (D) CBX4. (E) CBX5. (F) CBX6. (G) CBX7. (H) CBX8. *

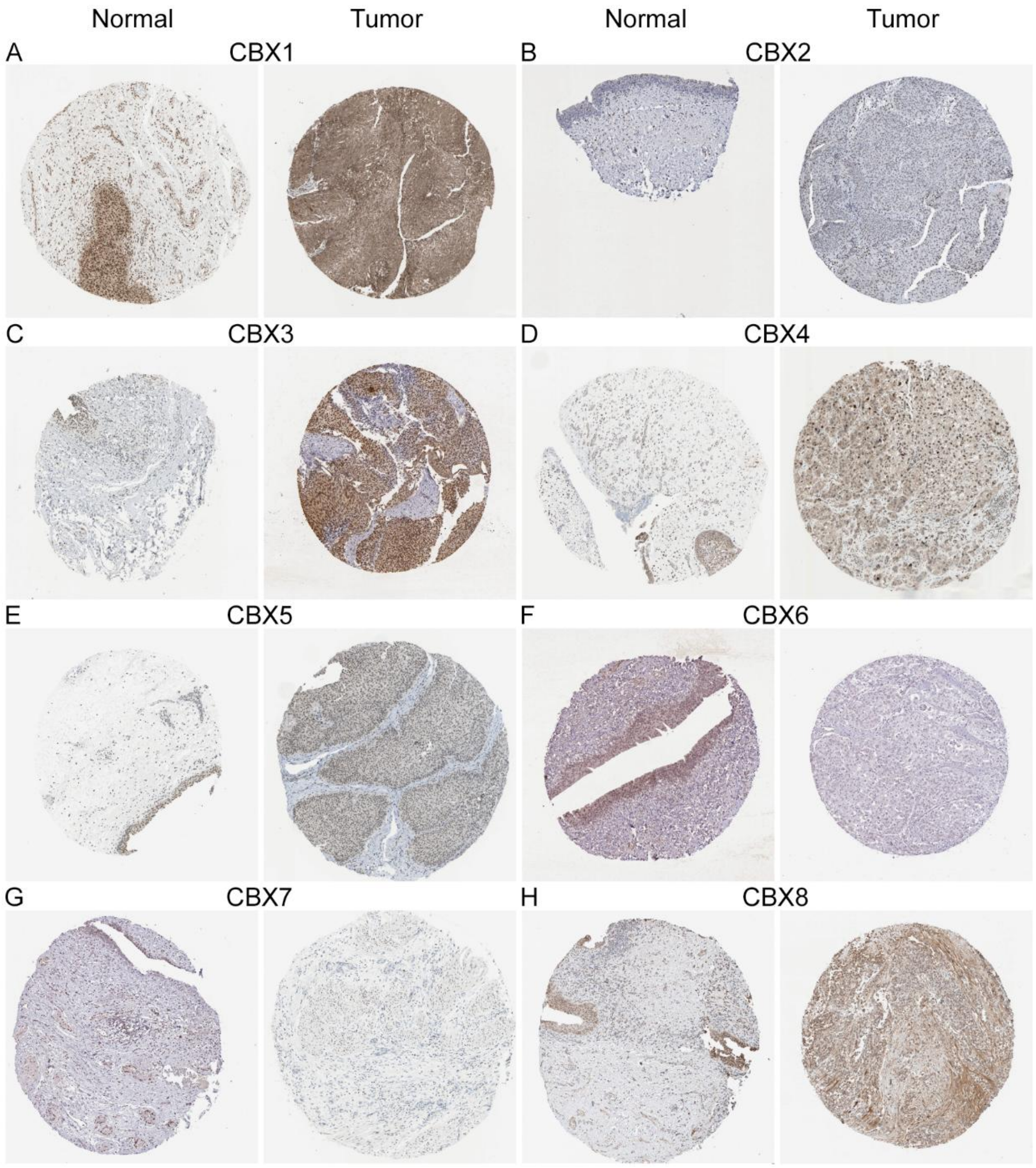

S

Figure S3. Immunohistochemical of CBXs family members on the Human Protein Atlas database. (A) CBX1; (B) CBX2; (C) CBX3; (D) CBX4; (E) CBX5; (F) CBX6; (G) CBX7; (H) CBX8. L, low expression; M, middle expression; H, high expression.

Mult_cox	p.value	Hazard Ratio(95% CI)
CBX1	0.63854	0.93036(0.68843,1.25733)
CBX2	0.52517	0.953(0.8215,1.10555)
CBX3	0.2876	0.82467(0.57806,1.17649)
CBX4	0.35461	1.15676(0.84983,1.57454)
CBX5	0.01112	1.33345(1.06782,1.66517)
CBX6	0.00894	1.27233(1.06214,1.52411)
CBX7	0.0017	0.69874(0.55857,0.87407)
CBX8	0.85533	0.96979(0.69739,1.34859)
Age	0.00018	1.03042(1.0144,1.0467)
Gender	0.36507	0.85135(0.60098,1.20601)
Race	0.52246	0.90441(0.66473,1.23051)
pTNM−stage	<0.0001	1.69912(1.38021,2.09171)

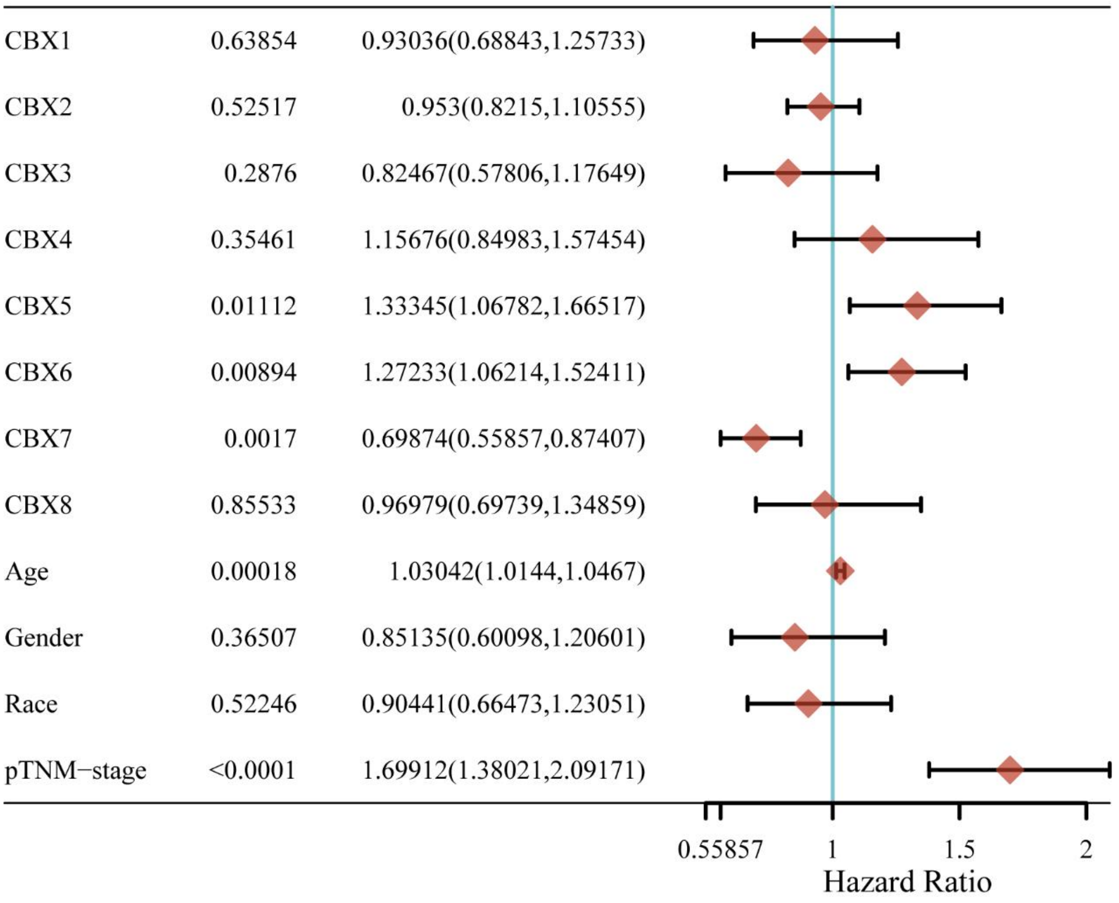

Figure S4. Multivariate COX regression analysis of CBXs and clinical factors by the Assistant of Clinical Bioinformatics website.
